# Supplementary figures and images for: Transformation of primary murine peritoneal mast cells by constitutive KIT activation is accompanied by loss of Cdkn2a/Arf expression
Source: Front Immunol. 2023 Mar 30;14:1154416. doi: 10.3389/fimmu.2023.1154416 (PMC10097954; doi:10.3389/fimmu.2023.1154416)

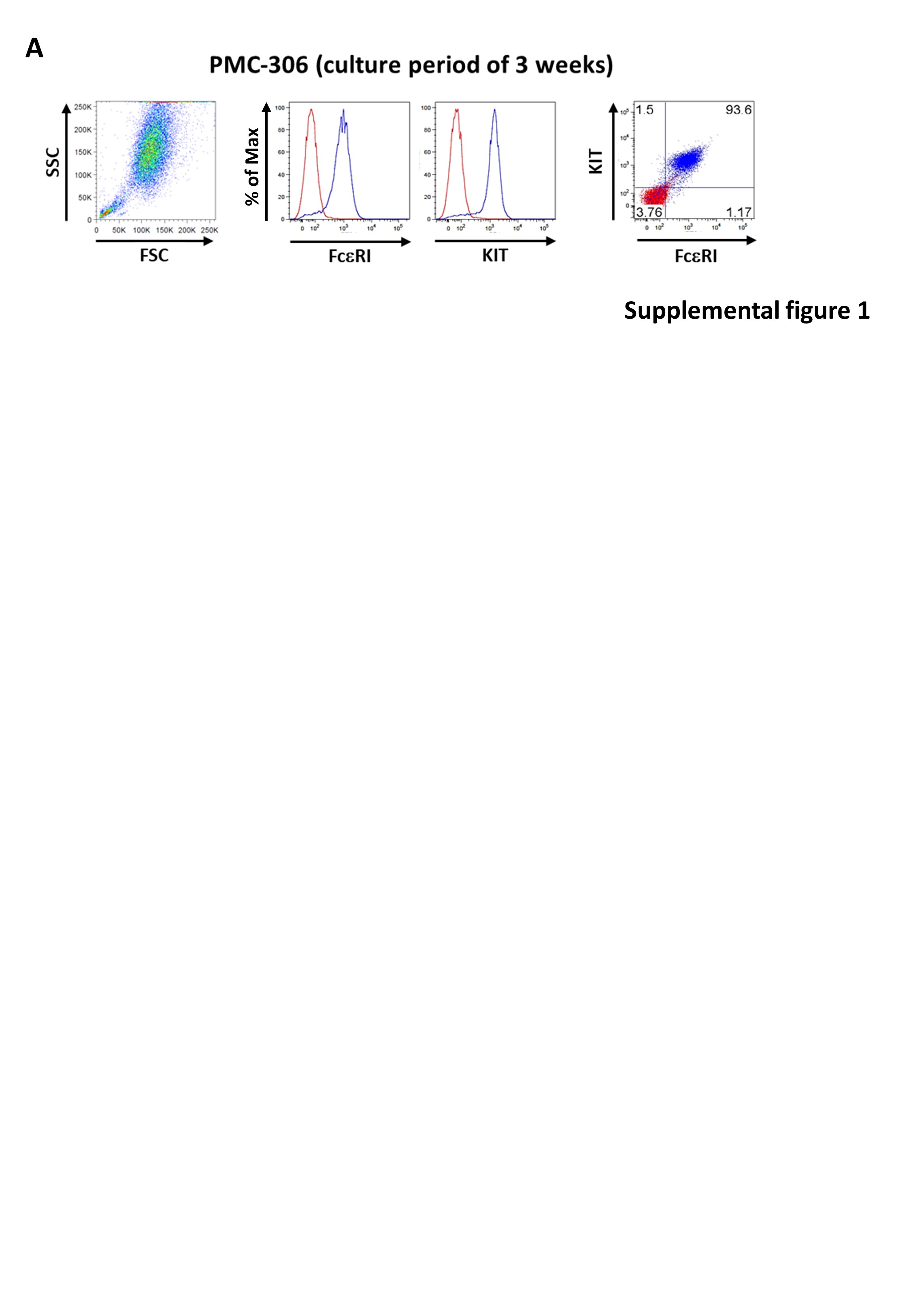

Supplement: Supplementary Figure 1 — MC surface marker expression of PMC-306 before and after immortalization. (A) Representative FACS analysis of PMC-306 cells before transformation after 3 weeks under regular PMC culture conditions. FSC/SSC dot plot shows a typical WT PMC population of 93.6% FcεRI and KIT double-positive cells (n=3). [file Image_1.jpeg]

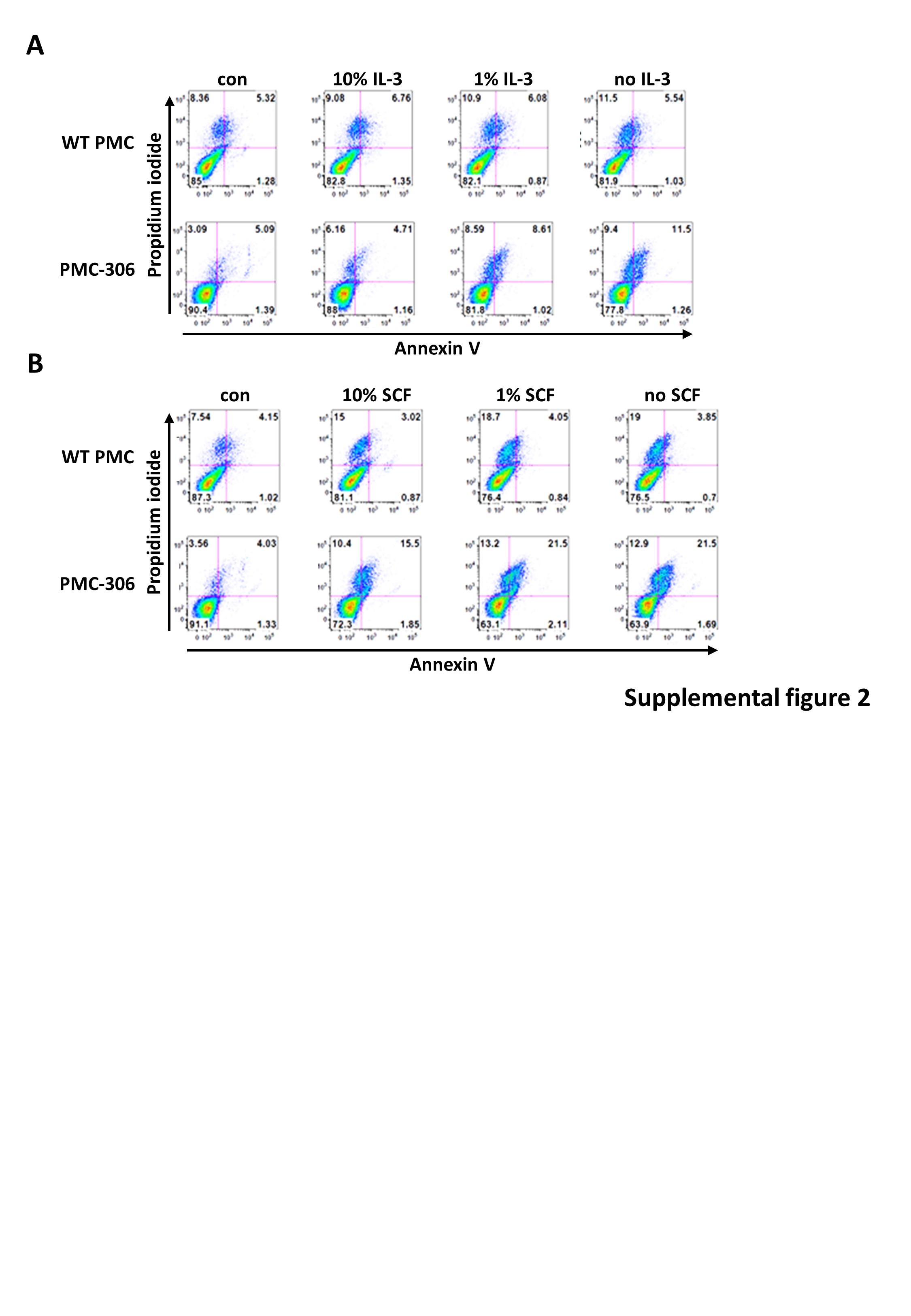

Supplement: Supplementary Figure 2 — Analysis of primary WT PMC and PMC-306 viability under cytokine deprivation conditions. Representative FACS dot plots showing Annexin V and propidium iodide positivity in primary PMCs and PMC-306 cells under IL-3 (n=3) (A) or SCF (n=3) (B) deprivation for 72 hours. The percentage of single positive, double positive, and double negative cells is provided in the associated gates. [file Image_2.jpeg]

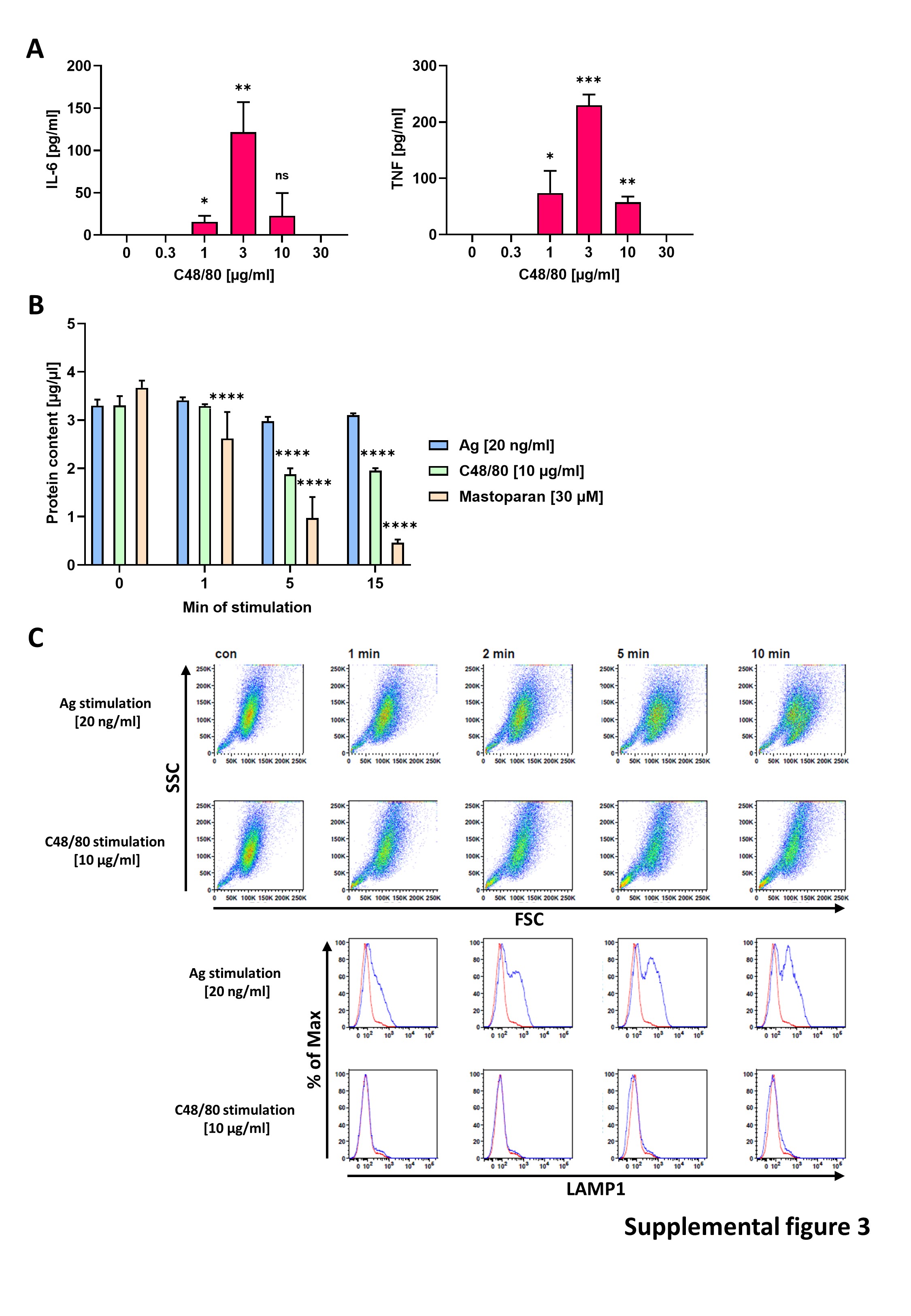

Supplement: Supplementary Figure 3 — Analysis of MRGPRB2 activation shows unconventional degranulation and impact on cellular integrity. (A) ELISA measurement of secreted IL-6 (left) or TNF (right) from PMC-306 cells stimulated with increasing concentrations of C48/80 (n=4). (B) BCA assay to determine the protein concentration in cell lysates of PMC-306 cells stimulated with either Ag [20 ng/ml], C48/80 [10 µg/ml] or Mastoparan [30 µM] for the indicated time points (n=3). (C) Representative flow cytometry dot plots and histograms of primary WT PMCs stimulated with either Ag [20 ng/ml] or C48/80 [10 µg/ml] for indicated time points and stained with anti-LAMP1 to determine granule externalization (n=3). Data are shown as mean +SD. (A) Ordinary one-way ANOVA followed by Dunnett multiple comparisons test. (B) Two-way ANOVA followed by Sídák multiple comparisons test. p>0.05 ns, *p<0.05, **p<0.01, ***p<0.001, ****p<0.0001. Stars indicate the significance level relative to the respective controls. [file Image_3.jpeg]

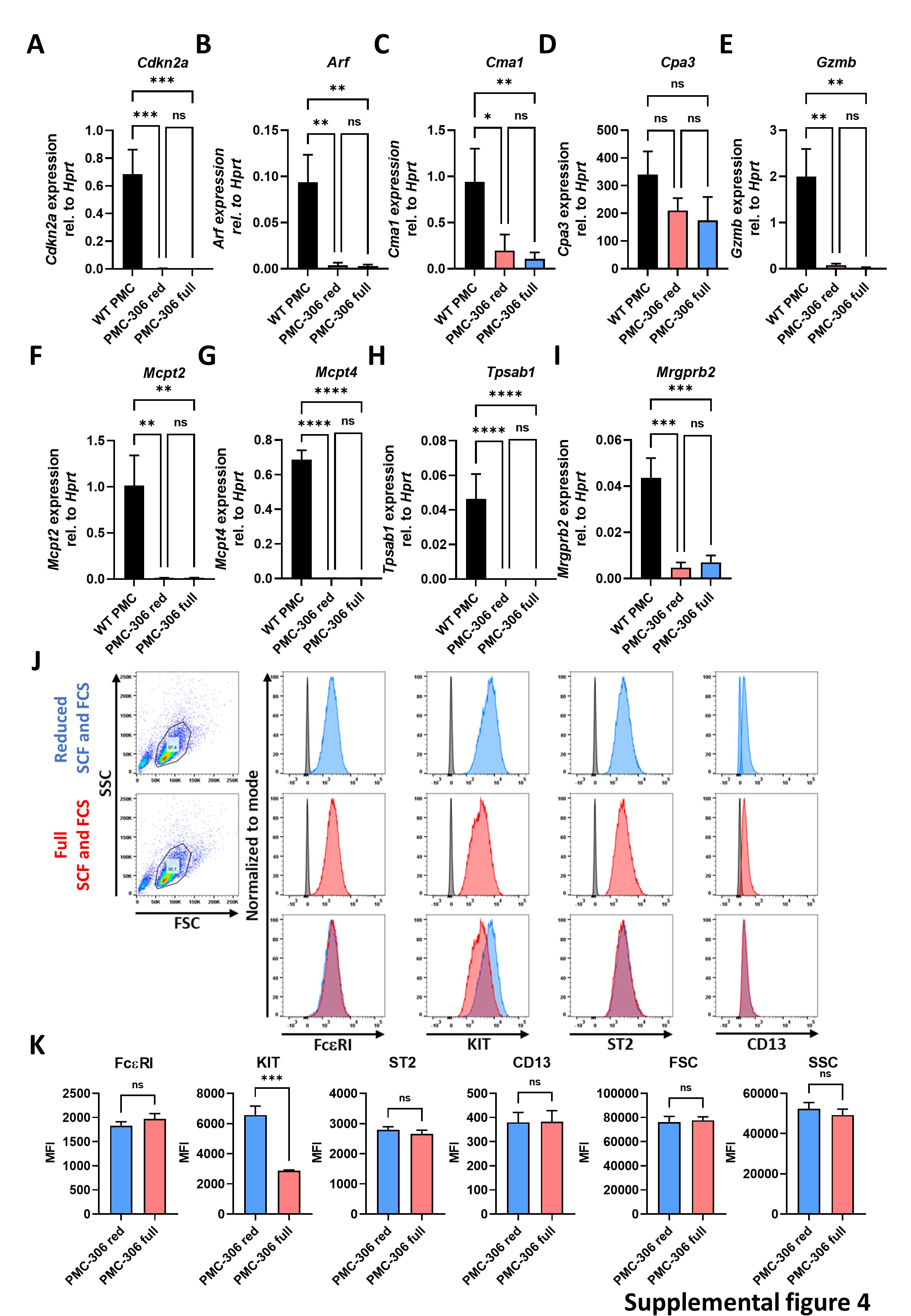

Supplement: Supplementary Figure 4 — Different culture conditions do not affect the general PMC-306 phenotype. (A–I) PMC-306 cells were cultivated either under reduced conditions with 10% FCS and 5 ng/ml SCF or primary PMC conditions (full, 15% FCS and 20 ng/ml SCF) to analyze potential effects on different culture conditions on gene expression. Primary WT PMCs cultivated under normal PMC conditions served as reference. mRNA expression of Cdkn2a (A), Arf (B), Cma1 (C), Cpa3 (D), Gzmb (E), Mcpt2 (F), Mcpt4 (G), Tpsab1 (H) and Mrgprb2 (I) were analyzed by qPCR. Expression of the respective genes of interest was quantified by the delta CT method using Hprt as a housekeeping gene (n=3). (J) Representative FACS dot plots and normalized histograms showing expression of FcεRI, KIT, ST2 and CD13 in PMC-306 cells cultivated either under reduced (blue) or full medium (red) conditions. The upper two panels show frequency distributions relative to an unstained control (grey) while the lower panel shows frequency distribution comparing reduced and full medium conditions. (K) Quantification of the FACS surface marker analysis depicted in (J) with MFIs showing staining intensities for the respective surface markers as well as FSC and SSC parameters (n=3). (A–I) One-way ANOVA followed by Dunnett multiple comparisons test. (K) Unpaired, two-tailed Student’s t-test with Welsh’s correction. Data are expressed as mean +SD. p>0.05 ns, *p<0.05, **p<0.01, ***p<0.001, ****p<0.0001. [file Image_4.jpeg]

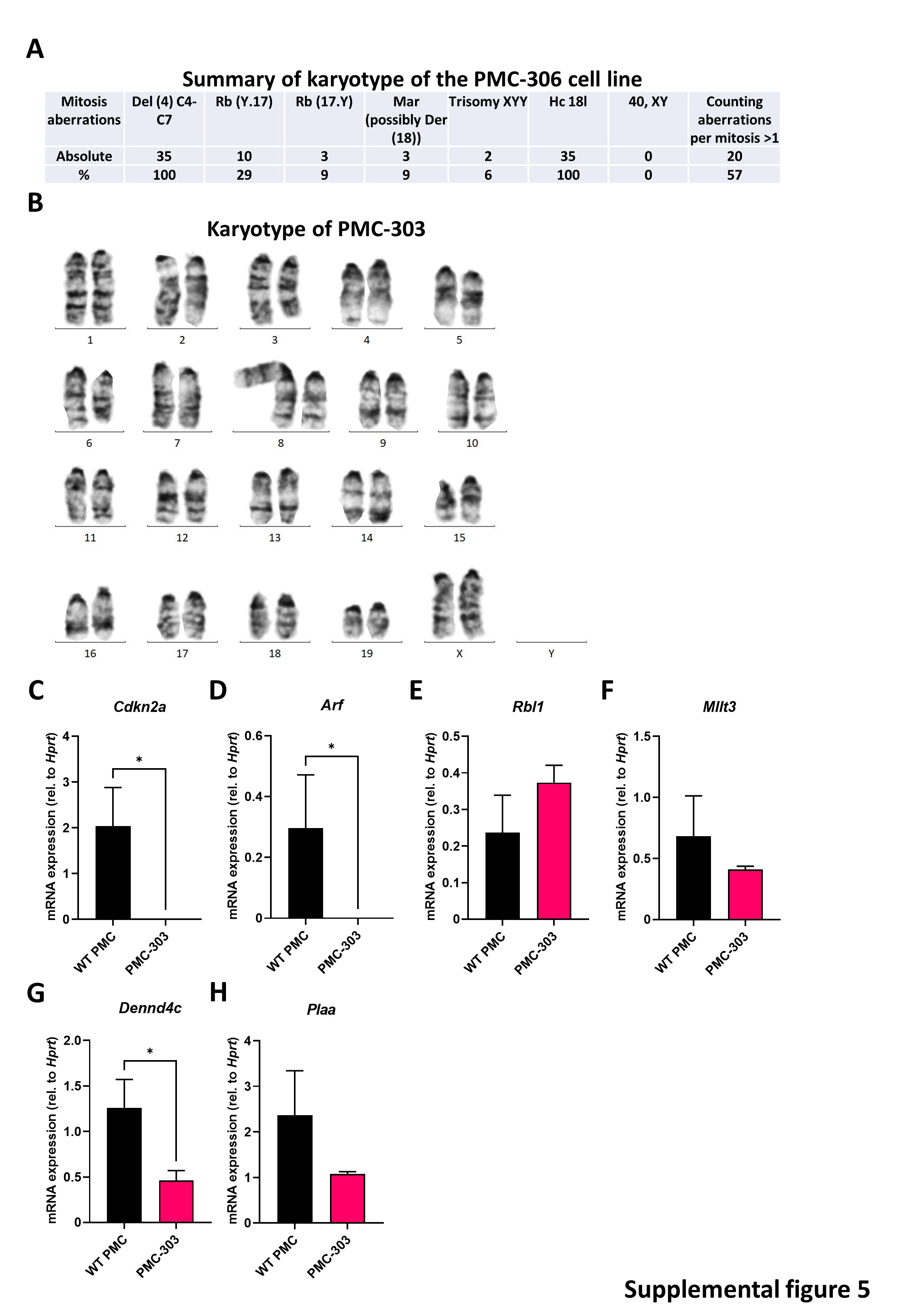

Supplement: Supplementary Figure 5 — Cytogenetic and gene expression analysis of the independently transformed PMC cell line PMC-303. (A) Table of all discovered cytogenetic aberrations of mitotic chromosomes of the PMC-306 cell line with associated frequencies. (B) Representative G-banded karyotype of the PMC-303 cell line with trisomy 8 due to a homologous Robertsonian translocation of chromosome 8 (Rb(8.8)). (D–H) RT-qPCR analysis of genes encoded within in the region of chromosome 4, which is deleted in the PMC-306 cell line (Chr4 qC4-qC7). Gene expression of Cdkn2a, Arf, Rbl1, Mllt3, Dennd4c and Plaa were measured in PMC-303 cells in comparison to primary WT PMCs (n=3). Data are expressed as mean +SD. Unpaired, two-tailed Student’s t-test with Welsh’s correction. *p<0.05. [file Image_5.jpg]

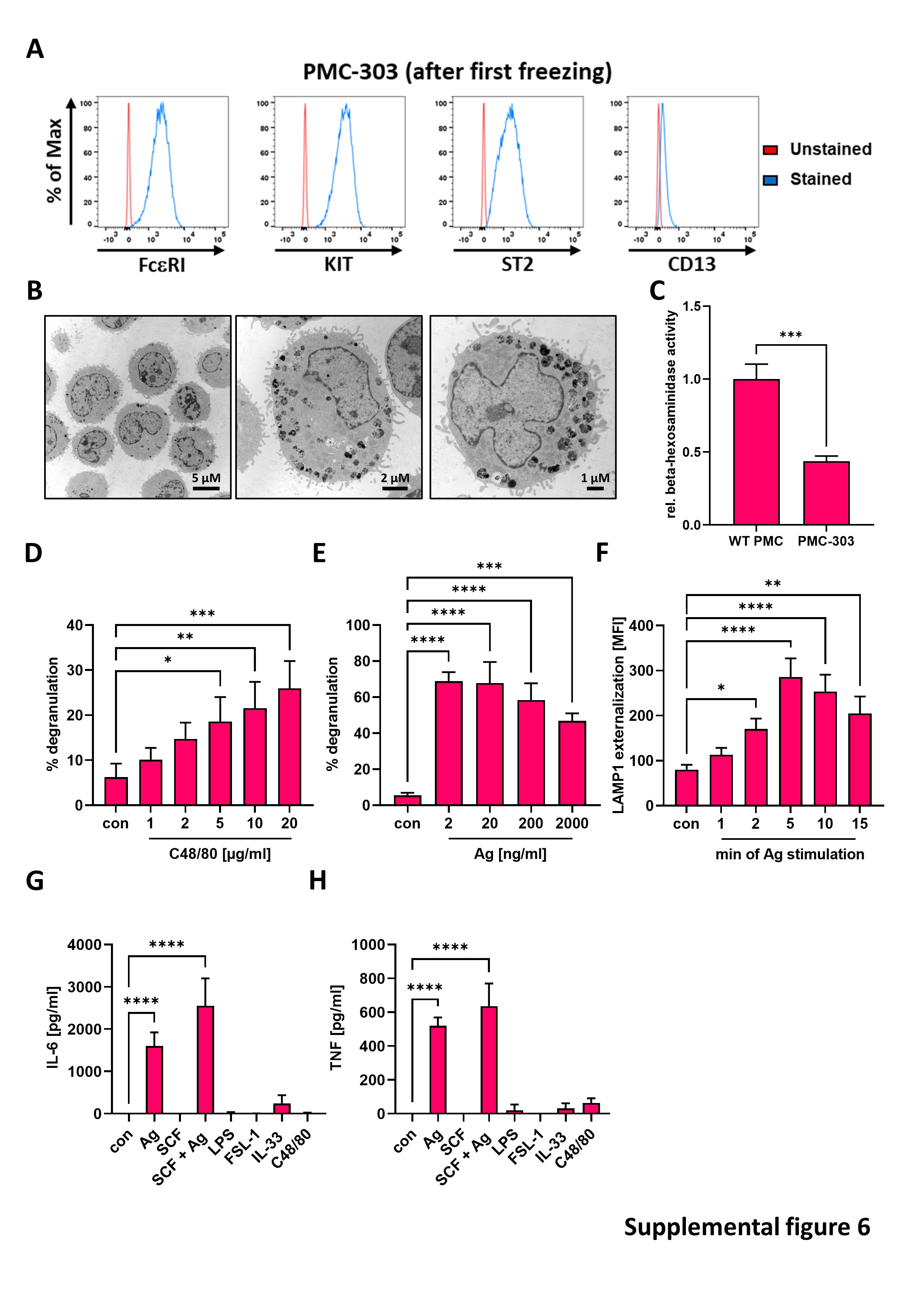

Supplement: Supplementary Figure 6 — Characterization of the PMC-303 cell line. (A) Representative FACS histograms showing surface localization of FcεRI, KIT, ST2 and CD13 in PMC-303 cells after freeze-thaw relative to an unstained control (n=3). (B) Representative electron micrographs showing the ultrastructure of PMC-303 cells. (C) Quantification of the relative β-hexosaminidase activity in lysates of primary PMCs and PMC-303 cells determined by an enzymatic assay to quantify the amount of cellular β-hexosaminidase expressed in primary PMCs and PMC-306 cells (n=3). (D) Determination of the degranulation of PMC-303 cells in response to indicated concentrations of C48/80 assessed by β-hexosaminidase release assay (n=3). (E) Determination of the degranulation of PMC-303 cells in response to indicated concentrations of Mastoparan assessed by β-hexosaminidase release assay (n=3). (F) FACS analysis of PMC-303 cells stimulated with Ag [20 ng/ml] for indicated time points to determine externalization of LAMP1 externalization. Bar graphs shown MFIs for LAMP1 for each time point (n=3). (G) The pro-inflammatory cytokine production of PMC-303 cells to Ag (20 ng/ml) in comparison to other stimuli (SCF: 100 ng/ml, SCF+Ag: 100 ng/ml and 20 ng/ml, LPS: 1µg/ml, FSL-1: 1 µg/ml, IL-33: 10 ng/ml, C48/80: 10 µg/ml) was determined by IL-6 ELISA (n=3). (H) Same experiment as in (G), but secreted amounts of TNF were determined by ELISA (n=3). Data are expressed as mean +SD. (C) Unpaired, two-tailed Student’s t-test with Welsh’s correction. (D–H) One-way ANOVA followed by Dunnett multiple comparisons test. Stars indicate significance within one group relative to control. p>0.05 ns, *p<0.05, **p<0.01, ***p<0.001, ****p<0.0001. [file Image_6.jpg]

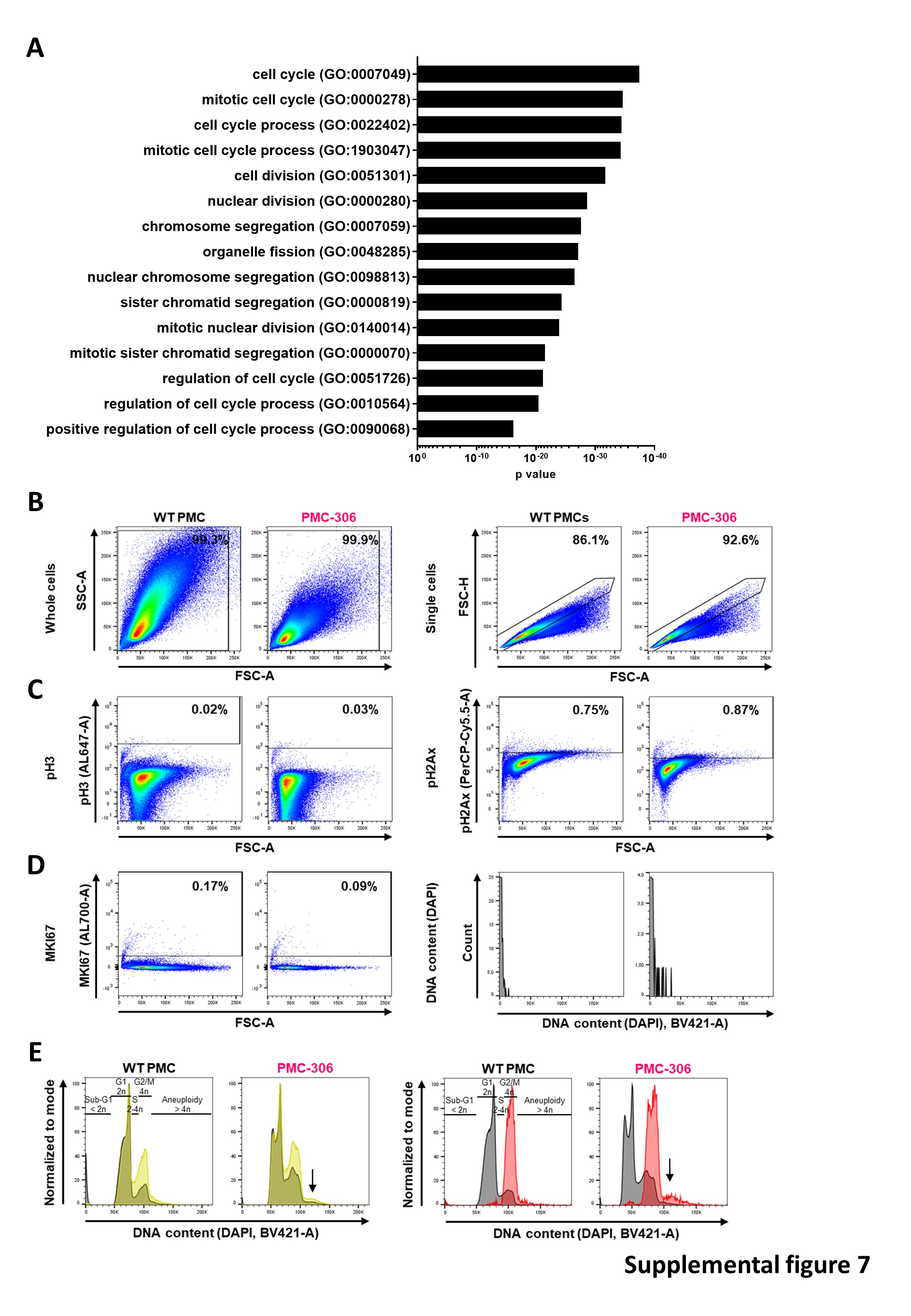

Supplement: Supplementary Figure 7 — GO Term analysis of the NGS data set, gating strategy and background determination for flow cytometry (FACS) and analysis of aneuploidy. (A) For the GO term enrichment analysis all genes regulated at least 5-fold between primary WT PMCs and PMC-306 cells were included. The top 15 GO terms with the lowest p values are depicted. GO term analysis was performed using the gene ontology knowledgebase server (http://geneontology.org/) (B–D) Gating strategy and background determination for the transformed PMC-306 line (red) and primary WT PMCs (black). (B) Left panels: Forward versus side scatter (FSC vs. SSC) gating for identification of cells of interest. FSC indicates cell size, SSC indicates cell granularity. Right panels: FSC-A vs. FCS-H gating for identification of single cells. (C, D) Unstained controls, which were not incubated with antibodies to assess background for staining against phospho-histone 3 (AL647-A, pH3) and phospho-histone H2Ax (pH2Ax, PerCP-Cy5.5-A) (C), MKI67 (AL700-A) and DAPI (DNA content, BV421-A) (D). (E) Representative histograms of primary WT PMCs and PMC-306 cells stained with an antibody directed against MKI67 (AL700-A) and phospho-histone H3 (pH3, AL647-A). Cellular DNA content was determined by DAPI staining (BV421-A) for assignment to the distinct cell cycle phases as follows: SubG1:< 2n DNA content; G1: 2n; S: 2-4n; G2/M: 4n; Aneuploidy: > 4n. Representative overlay of FACS-histograms of DNA content in all single cells (black) versus MKI67+ (yellow, left) or pH3+ (red, right) single cells (normalized to mode). Black arrows indicate MKI67+ and pH3+ cells with a DNA content above 4n (aneuploidy) (n=3). [file Image_7.jpg]
